# The intergenerational transmission of partnering

**DOI:** 10.1371/journal.pone.0205732

**Published:** 2018-11-13

**Authors:** Claire M. Kamp Dush, Rachel Arocho, Sara Mernitz, Kyle Bartholomew

**Affiliations:** 1 Department of Human Sciences, The Ohio State University, Columbus, OH, United States of America; 2 Carolina Population Center, The University of North Carolina at Chapel Hill, Chapel Hill, NC, United States of America; 3 Population Research Center, University of Texas, Austin, TX, United States of America; 4 Pay Tel, Greensboro, NC, United States of America; Centre d'Estudis Demografics, SPAIN

## Abstract

As divorce and cohabitation dissolution in the US have increased, partnering has expanded to the point that sociologists describe a merry-go-round of partners in American families. Could one driver of the increase in the number of partners be an intergenerational transmission of partnering? We discuss three theoretical perspectives on potential mechanisms that would underlie an intergenerational transmission of partnering: the transmission of economic hardship, the transmission of marriageable characteristics and relationship skills, and the transmission of relationship commitment. Using the National Longitudinal Survey of Youth 1979 Child and Young Adult study (NLSY79 CYA) and their mothers in the National Longitudinal Survey of Youth 1979 (NLSY79), we examined the intergenerational transmission of partnering, including both marital and cohabitating unions, using prospective measures of family and economic instability as well as exploiting sibling data to try to identify potential mechanisms. Even after controlling for maternal demographic characteristics and socioeconomic factors, the number of maternal partners was positively associated with offspring’s number of partners. Hybrid sibling Poisson regression models that examined sibling differential experiences of maternal partners indicated that there were no differences between siblings who witnessed more or fewer maternal partners. Overall, results suggested that the transmission of poor marriageable characteristics and relationship skills from mother to child may warrant additional attention as a potential mechanism through which the number of partners continues across generations.

## Introduction

Stable romantic unions, including marriage and cohabitation, are linked to better mental and physical health for both adults and children [1]. However, maintaining such unions can be difficult; half of first cohabiting unions dissolve within three years [2] and half of first marriages dissolve within twenty years [3]. After a union dissolves, most individuals repartner [4]. Repartnering is defined as remarriage or cohabitation after a union dissolution [5]. Unions formed through repartnering are more likely to end than first unions [6, 7], creating additional opportunities for more repartnering. Repartnering has grown such that 9% of American children live in households with a cohabiting or married stepparent [8], 55% of whom live with a married step-parent and 45% with a cohabiting step-parent [9], and further, 11% live with half or step-siblings [10]. Second unions may have increased health benefits compared to first [11], and children whose mothers repartner may grow up in a household with increased economic wellbeing [12]. At the same time, due to their increased rates of dissolution, repartnered unions can increase family instability with potentially negative effects for both adults and children [13].

Partnering is operationalized as the number of partners one experiences and includes multiple processes including initial union formation, divorce or dissolution, and repartnering. Partnering behaviors may be transmitted across generations. Children of divorce are more likely to divorce themselves, increasing the opportunity for repartnering [14]. Cohabitation rates have doubled over the past 25 years [15] and the proportion of children born to cohabiting parents has also grown [16]. A majority of children who experience maternal cohabitation will also experience its dissolution [17], opening the door to more partners entering their lives. Little work has examined the intergenerational transmission of partnering including cohabitation dissolution as well as divorce. A recent study by Amato and Patterson [18] found an intergenerational transmission of partnering using retrospective data but Amato and Peterson were unable to account for concurrent changes in family economic hardship that could explain this intergenerational transmission.

Using the National Longitudinal Survey of Youth 1979 Child and Young Adult study (NLSY79 CYA) and mothers in the National Longitudinal Survey of Youth 1979 (NLSY79), we examine the intergenerational transmission of partnering, including spouses and cohabitating partners. Three theoretical perspectives guided our analyses. The economic hardship perspective suggests that economic hardships resulting from divorce drives the intergenerational transmission of partnering. Economic hardship causes more conflict in intimate relationships [19], rendering the unions of the poor less stable [2] and thus increasing the opportunity for repartnering. Further, poor single mothers may be more motivated to enter into a new union to secure additional economic capital for their children [12], although second unions tend to dissolve quickly [20] and may lead to additional partnering.

The intergenerational transmission of marriageable characteristics and relationship skills perspective [14, 21] argues that heritable maternal characteristics as well as maternal relationship skills and behaviors undermine union success and increase the likelihood of repartnering. Manning, Trella [21] have argued that both men and women may not be “marriageable”, that is, may have characteristics that make them undesirable partners, including depression, substance use, and poor economic prospects. Some of these characteristics may be passed from mother to child. For example, mothers who are depressed are more likely to have children with elevated internalized problems [22]. Maternal personality traits such as the ability to trust others are also evident in their offspring [23]. Mothers who use substances in adolescence and early adulthood are more likely to have children who do so [24]. Parents’ relational and conflict behavior are associated with their own children’s relational behavior in adolescence and adulthood [25, 26] Thus, according to this perspective, mothers pass their marriageable characteristics on to their offspring, and these characteristics and behaviors, not the number of maternal partners itself, drive the intergenerational transmission of partnering. Extending this argument, siblings who share genes as well as a family environment should report similar levels of partnering, even if one sibling experienced more maternal partners because they were born during their mother’s first union compared to a later sibling who experienced fewer partners because they were born during a later union.

The intergenerational transmission of commitment perspective suggests that witnessing a repartnering, rather than the economic hardship, maternal characteristics, or family environment associated with the repartnering, leads offspring to have less stable unions [14] and thus a higher number of partners. This perspective proposes that witnessing commitments being broken teaches children that unions do not need to last a lifetime, and that new unions can be formed that may better meet an individual’s needs. Thus a sibling who witnesses the entry of a new partner should have a significantly higher likelihood of repartnering compared to the sibling who did not. Given that multipartner fertility—having children with new partners after having had children in past relationships—has led to increased family complexity in the US, this is not an uncommon situation; 11% of children live in blended families with half- or step- siblings who do not share the same biological parents [10]. We are able to test the following competing hypotheses based on these three theoretical perspectives because the NLSY79 CYA includes all children born to the mothers in the NLSY79.

### Theoretical perspectives on the intergenerational transmission of partnering

#### Economic hardship perspective

Both divorce and cohabitation dissolution have negative financial consequences [27]. Once a mother repartners, economic resources may increase [28], but poverty rates for economically disadvantaged divorced mothers exceed those of never-married mothers [29]. The economic hardship perspective argues that the family financial difficulties experienced by young adults who experienced their mothers’ repartnering is primarily responsible for the negative outcomes that these young adults experience, namely their own proclivity to partner multiple times, through four mechanisms. First, the economic consequences of dissolution may have serious implications for young adult development. Individuals who were raised in families with fewer economic resources have poorer socioemotional, cognitive, and behavioral development in childhood [30] and have lower academic achievement in adulthood [31], which could render these young adults less attractive partners in the marriage market and once in a union [21].

Secondly, socioeconomic status transmits across generations [31] and the economic stress that results from economic hardship is one of the most common and significant causes of relationship conflict [19]. Unsurprisingly, economic stress is associated with an increased odds of union dissolution [32] and lower odds of moving from cohabitation to marriage [19], opening the door for more partners if the union dissolves.

Another mechanism may be the young adult’s age at first union formation. Young adult offspring who experience their mother dissolving and forming unions leave home and assume adult roles and responsibilities earlier, including entering unions [33]. In contrast, offspring with more resources may delay entering a union because they are in college and perceive these years as a time for self-development [34]. Younger ages at marriage and cohabitation are associated with elevated union instability and increased opportunity for partnering [35]. Arnett [36] has argued that identity development among young adults in developed countries has been delayed and that young adults now experience a new developmental period called emerging adulthood. Early unions may also be unstable because they were formed during emerging adulthood prior to identity development.

Finally, offspring from more advantaged backgrounds often receive resources from their parents that allow them to delay their entrance into coresidential unions, such as help with college tuition and residential costs [37, 38]. In contrast, single mothers have fewer resources to provide [39]. If the mother repartners, she may have difficulty providing resources because stepparents and remarried biological parents are less likely to approve of support for adult children [40], leaving the offspring with fewer resources and a greater incentive to enter union(s) that may then be rendered more unstable due to economic stress. Indeed, cohabitors often cite saving money as a motivation for cohabitation [41].

*Hypothesis 1*. Based on the economic hardship perspective, we hypothesized that a significant association between maternal and offspring partnering would either become nonsignificant or be reduced in magnitude following the addition of maternal employment, education, and poverty to the model.

#### Intergenerational transmission of marriageable characteristics and relationship skills

A second mechanism is the intergenerational transmission of marriageable characteristics (i.e. an agreeable disposition, greater educational attainment) and relationship skills (i.e. communication and conflict resolution styles). Family scholars have long argued that some individuals are more “marriageable” than others; that is, that some individuals possess characteristics that make them attractive potential romantic partners [21]. Through both genetics and the environment, parents play an important role in shaping offspring’s marriageable characteristics. For example, children of depressed mothers have elevated interpersonal dysfunction, negative cognitions, and poor emotion regulation that increase their risk of adult depressive symptoms [42, 43]. Individuals with more depressive symptoms have less stable unions [44]. Personality traits also heritable, hence maternal personality traits that undermine union stability may be shared by their offspring [45]. Individuals who are more agreeable, extraverted, conscientious, and less neurotic tend to be desirable romantic partners [46] and have more stable unions [47]. If the intergenerational transmission of marriageable characteristics is driving the intergenerational transmission of partnering behavior, then we would expect siblings to be similar to one another in their own levels of partnering due to shared genes and environment, even if they had different experiences with their mothers’ partnering.

Amato and DeBoer [14] argued that there is also an intergenerational transmission of relationship skills. They suggest that young adults learn relationship skills by watching their parents interact and through their own interactions with their parents and those that learn poor skills from their parents are more likely to have unstable relationships [14, 25]. Couples that divorce have poorer communication styles, less provision or receipt of social support, and more undermining and destructive conflict [48, 49], all of which may be witnessed, and learned, by their children. Indeed, young adults who witnessed a parental divorce exhibit poorer relationship functioning than those whose parents did not divorce [26]. Less research has been conducted on the relationship functioning predictors of cohabitation dissolution, but given the high levels of instability among cohabiting unions with children [17], children in cohabiting families may be more likely than children in married families to witness negative relationship behaviors that, if learned, could make their own relationships less stable [50, 51].

*Hypothesis 2*. Based on the intergenerational transmission of marriageable characteristics and relationship skills perspective, we hypothesized that there would be no sibling differences in the significant association between maternal and offspring partnering.

#### The intergenerational transmission of commitment

The intergenerational transmission of commitment perspective suggests that witnessing their parents’ partnering contributes to young adult offspring union instability [14]. Specifically, young adults who observe their parents breaking a commitment and repartnering learn that committed relationships can be dissolved and new unions can be formed that may better meet an individual’s needs. Witnessing repartnering may thus weaken their commitment to their union particularly if relationship quality declines [14]. Many unions decline in relationship quality over time [52, 53], thus it may be inevitable that partners experience some disillusionment. This perspective argues that young adults who observe their parent in a stable union will be less likely to dissolve any union themselves, even if their parents had an earlier, unstable union prior to that young adult’s birth. Those young adults whose parents divorced are more likely to question the stability and permanence of their own relationships [14, 54], even when controlling for their own relationship’s quality. Interestingly, the children of divorce generally do not believe that marriage should be avoided [55], and those that want to avoid marriage do not avoid cohabitation [56]. When comparing the intergenerational transmission of relationship skills and commitment perspectives as it relates to divorce, Amato and DeBoer [14] found more support for the transmission of commitment perspective. The offspring of parents who dissolved less distressed marriages were more likely to divorce and offspring who grew up with parents whose relationships were highly distressed but stable were less likely to divorce [14]. A final mechanism of the transmission of commitment could be through maternal cohabitation. Cohabiting unions with children are less committed than marriages [57], thus offspring who experience maternal cohabitation may enter their own unions with less commitment and be more likely to dissolve their union and then repartner.

*Hypothesis 3*: Based on the intergenerational transmission of commitment perspective, we hypothesized that siblings who experienced more maternal partners would report having a greater number of partners than their siblings who experienced fewer maternal partners.

#### Previous research related to the intergenerational transmission of partnering

There is a robust body of research on the intergenerational transmission of divorce. Young adults who experience a parental divorce are significantly more likely to divorce themselves [14, 58]. There has been some debate in the literature about whether the intergenerational transmission of divorce has changed over time, but when the exposure to the risk of divorce is considered, Li and Wu [59] found no change over time in the intergenerational transmission of divorce. A recent paper by Amato and Patterson [18] examined retrospective data on maternal union instability, including cohabitation and marriage, measured when the offspring was an adolescent, and its association with offspring union instability. Amato and Patterson [18] found evidence for an intergenerational transmission of instability even after controlling for several variables including family income at age 16, maternal education, maternal religiosity, parent-child closeness, offspring depression and delinquency, offspring educational attainment, and the number of sex partners. Their results did not support the economic hardship perspective, but their lack of prospective and concurrent data on economic hardship was an important limitation.

#### Selection

Selection may play a significant role in the intergenerational transmission of partnering. African-American and Hispanic women are more likely to have births outside of marriage and cohabitation [60] which sets the stage for new partners to come into the mother and children’s lives. Most African-American women are single at the time of their nonmarital birth whereas Hispanic women are more likely to give birth within a cohabiting union [60]. Cohabiting unions with children face an increased likelihood of dissolution [17]. Thus, we control for maternal family structure at birth, maternal race, as well as the offspring’s own parental status. Maternal age at child’s birth is also associated with a variety of offspring outcomes [58] thus we also control for it. Finally, male and female young adult offspring may respond differently to parental divorce [61] and older young adult offspring will have more opportunity to exit and enter successive unions. Therefore, gender and age of young adults at the last recorded interview were also included in each model.

## Method

We use the National Longitudinal Survey of Youth 1979 (NLSY79) and the National Longitudinal Survey of Youth 1979 Child and Young Adult (NLSY79 CYA) datasets. The NLSY79 is a longitudinal sample consisting of 12,686 young men and women ages 14–22 when first interviewed in 1979 and was nationally representative in 1979. Data were collected annually until 1994 and biennially thereafter. The NLSY79 CYA data were collected biennially from all biological offspring of the women in the NLSY79 beginning in 1986. Through 1992, all data was mother-reported, but beginning in 1994, offspring who were aged 15 and older completed a lengthy interview similar to the NLSY79 interview. Because offspring age into the young adult sample, the sample over-represents offspring born to the youngest mothers. By 2012, 76% of all maternal offspring in the NLSY79 CYA dataset had been interviewed as a young adult at least once (*n* = 7999). Of these young adults, we retained those who were at least 18 and for whom we had complete maternal partnership data to that point, *n =* 7152 (*n =* 3272 mothers). Of these, 91% had been interviewed at age 20, 60% at age 25, and 24% at age 30. The average age at first cohabitation for this cohort is 21.8 and 23.5 for women and men respectively [62]. Of this analytic sample of 7152, 87% (*n =* 6246) of offspring had at least one sibling in the sample (2366 total sibships).

### Variables

Descriptive statistics for the full sample and the sibling sample are reported in Table 1.

**Table 1:** Means, standard deviations, and percentages for full sample and siblings only sample.

	Full Sample				Siblings Only		
Variable	Mean	%	SD	% Missing	Mean	%	SD
Offspring Partners	1.16		1.35	0%	1.19		1.37
Maternal Partners (Total)	1.73		1.24	0%	1.74		1.26
Maternal Cohabiting Partners	0.95		1.19	0%	0.95		1.21
Maternal Marital Partners	1.19		0.70	0%	1.19		0.69
Maternal Race (Reference: Non-Hispanic Non-Black)							
Hispanic		22%		0%		23%	
Black		34%		0%		35%	
Maternal Age at Offspring Birth	23.74		4.83	0%	23.64		4.80
Maternal Marital Status as Offspring Birth (Reference: Married)							
Cohabiting		6%		2%		6%	
Single		31%		2%		31%	
Percentage of Childhood Exposed to Marriage	0.68		0.37	0.1%	0.69		0.37
Percentage of Childhood Exposed to Cohabitation	0.07		0.16	0.1%	0.07		0.16
Maternal Education (Reference: High School)							
Less than High School		15%		3%		16%	
Some College		26%		3%		26%	
College		9%		3%		8%	
More than College		6%		3%		5%	
Percentage of Childhood in Poverty	0.38		0.38	59%	0.40		0.39
Percentage of Childhood Mother Worked Full Time	0.42		0.33	0%	0.40		0.32
Percentage of Childhood Mother Worked Part Time	0.29		0.22	0%	0.30		0.22
Offspring Male		49%		0%		49%	
Offspring is a Parent		45%		0.1%		46%	
*n*	7,152				6,246		

#### Maternal total partners

The NLSY79 tracked respondent cohabiting partners and spouses across time. Partnership status was measured at the time of interview by self-reported marital and cohabitation status, and spouses and live-in partners were denoted in a household roster. At every wave, dates of up to three marital transitions since the last interview were collected, including divorce, marriage, and widowhood, even if a marriage began and ended between waves. Marriages that started before 1979 were retrospectively reported by date. Beginning in 1994, mothers reported if they had cohabited with the spouses they were currently married to, and when they had begun this cohabitation. In addition, unmarried respondents who reported living with a partner defined the beginning date of that union. Starting in 2000, beginning and end dates of cohabiting unions that were at least three months long were collected, even if the cohabitation started and ended between waves. We created an overall measure of the total number of maternal partners from these data. Because some relationship transitions were unclear (i.e. dates were missing, relationships ended and then later continued, and relationships overlapped), some relationships may have been missed and thus our measure may be an undercount. For each offspring, we generated a total maternal partner count that captured the number of partners each offspring had been exposed to since the year of their birth, including partnerships at the time of the child’s birth. Maternal total partner counts ranged from 0–9 (*M* = 1.73, *SD* = 1.24). We also created separate measures of maternal partners by marital status.

#### Offspring total partners

Offspring total partner number was assessed in the NLSY79 CYA for years 1994 to 2012 using several variables. The highest reported value for the item asking, “How many marriage or partner relationships have you been involved in?” was used for years 1994 through 1998. Two datum were recoded to missing, as their reported number of partners was so high as to likely represent mischievous responses (14 and 38 partners added between interviews). After 1998, this item was dropped from the survey. For 2000 through 2012, a combination of items determined the number of partners. First, all participants who consistently indicated “Never Married” as their marital status and never reported cohabiting were coded as having no partners. Second, all participants who ever marked married, separated, cohabited, divorced, or were widowed were counted as having a partner. Third, those who indicated that they were no longer with their spouse or partner from a previous wave, and were currently cohabiting or married, were counted as having an additional partner. Fourth, those who reported that they had cohabited with or married an uncounted partner between waves were coded as having an additional partner. The cumulative measure ranged from 0–17 (*M* = 1.05, *SD* = 1.32).

#### Controls

*Maternal race/ethnicity* was coded as Hispanic, Black, and Non-Black, Non-Hispanic. *Maternal education* at the child’s 18th year was measured using a series of categories including less than high school (less than 12 years completed education), high school (12 years,), some college (13 to 15 years), college (16 years), and more than college (17 or more years). *Maternal employment* was measured using mother’s retrospective weekly work accounts collected at each interview. Respondent’s weekly work hours were averaged over the year to produce measures of full time employment (more than 30 hours), part time employment (1–30 hours), or unemployment for that year (<1 hour per week on average). For each child, we recorded the percentage of interviews between ages 0 and 18 in which the mother reported each work status. *Maternal relationship status at childbirth* was coded as single, cohabiting, or married using the mother’s relationship status report at the child’s year of birth. For offspring who were born before 1979 (*n =* 814), relationship status was coded as married if the mother was reported married at that date, otherwise was left to missing and imputed. Those who were missing their marital or partnership status at the year of offspring’s birth were also imputed. This method may undercount maternal cohabitation at the time of birth. *Maternal age at childbirth* was measured in years (*M* = 23.74, *SD* = 4.83). *Offspring gender* was measured as 1 = Male. Offspring *parental status* was a dichotomous measure 1 = offspring reported they had had a child before the interview date. *Experience of Poverty* denoted the percentage of interviews between the child’s birth and age 18 that mothers reported a household income below the federal poverty line appropriate for the year and size of the household [63]. Following previous studies using these data [64], mothers who were missing the measure of income were coded as “in poverty” if they reported receiving at least $1 in governmental assistance through Assistance for Families with Dependent Children or Food Stamps. *Exposure to cohabitation* or *marriage* was coded as the percentage of interviews between the offspring’s birth and 18 years that mothers reported themselves to be married or cohabiting. These variables were coded missing if mothers were missing marital or partnership status at any interview and were imputed.

### Missing data

Missing data were imputed using augmented multiple imputation by chained equations in Stata14 [65]. In this method, each missing variable is the outcome in an appropriate regression on all other variables in the model and missing values are replaced with predicted values. Values were imputed over 60 datasets and analyses were conducted on each dataset separately with results pooled across models according to Rubin’s rules [66] using the MI suite of commands in Stata14.

### Analysis plan

Our dependent variable, offspring total partners, was a count variable with variance close to the mean (σ^2^ = 1.83, *M* = 1.16), thus we elected to use Poisson regression for analyses [67]. Because some offspring were observed longer than others, the offspring’s age at final observation was used as the exposure variable and fixed using the exp([var]) option for Poisson commands in Stata14 [68]. We first examined the association between offspring total partners and maternal total partners controlling for maternal race, age at childbirth, union status at childbirth, exposure to marriage and cohabitation, and offspring sex and parental status, and clustered the standard errors by mother to account for siblings within families. To test the economic hardship perspective, we then added socioeconomic controls, including maternal education, employment, and poverty status, to the model. We also separately examined the number of marital and cohabiting partners the mother experienced over the offspring’s life.

To test the intergenerational transmission of marriageable characteristics and relationship skills perspective, we exploited our sibling data and conducted between-within, also known as hybrid, sibling models [69]. Because the count of maternal partners was unique to each sibling’s lifetime, 438 sibships varied on their maternal partner exposure, allowing us to test differences between siblings while controlling for maternal characteristics that did not vary between siblings (such as personality). A between-within sibling model is similar to a traditional random and fixed-effects model which use each participant as their own control over time. The key difference is that rather than using each participant as their own control, each offspring is compared to their sibling; maternal characteristics that do not vary by sibling are thus accounted for in the model.

We elected to use the hybrid model for the ability to estimate the differences between siblings within mothers as well as offspring of different mothers. Our hybrid model used group-mean centered variables and included both the mean by mother and deviation from the mean by each sibling in the model. This model is a good test of the intergenerational transmission of marriageable characteristics and relationship skills perspective because we can measure variation both between children of different mothers and within families by comparing siblings to each other. By including the variable mean for all sibling-variant maternal variables, the between-within model [69] effectively positions the sibling model to exploit the variance in exposure to maternal partners between siblings.

## Results

To test our first hypothesis that the significant association between maternal and offspring partnering would become nonsignificant or be reduced after controlling for socioeconomic factors, we used clustered Poisson regression models (see Table 2). Overall, mothers who had more partners had offspring with significantly more partners. Poisson regression results can be interpreted by Incident Rate Ratios (IRR, calculated as exp^b^) and indicate the expected incident rate increase in the dependent variable from a one-unit increase in the independent variable. Without controlling for socioeconomic factors, each additional maternal partner was associated with a 6% incident rate increase in offspring partners. After controlling for socioeconomic factors, each additional maternal partner predicted a 5% incident rate increase in offspring partners. These coefficients for maternal total partners did not significantly differ between the two models when tested with Seemingly Unrelated Estimation using SUEST in Stata14 (*p* = .09). In models with and without socioeconomic controls, maternal marital and cohabiting partners were each significantly positively associated with offspring partners (see Table 3). Posthoc analyses indicated that no significant difference in the marital and cohabiting partners’ coefficients (*p* > .10). Compared to offspring with mothers who were non-Black and non-Hispanic, offspring with Hispanic or Black mothers had fewer partners. Offspring with mothers who were older at the offspring’s birth had significantly fewer partners, whereas offspring whose mothers were cohabiting at the offspring’s birth had significantly more partners. Male offspring and those who were parents themselves had significantly more partners. Offspring who spent a greater percentage of their childhood in poverty or with a mother who worked fulltime reported a great number of partners. In contrast, offspring whose mother had college education or more reported fewer partners. When data were examined by type of union, Hispanic ethnicity, maternal cohabitation at offspring birth, and maternal fulltime employment were no longer significant predictors of offspring partners, though the percentage of childhood exposed to cohabitation became a positive, significant predictor.

**Table 2:** Results of population poisson models with total maternal partners.

	Total Partners, No Economic Hardship				Total Partners, Economic Hardship			
	*b*	*SE*	*IRR*		*b*	*SE*	*IRR*	
Maternal Partners (Total)	0.06	0.01	1.07	***	0.05	0.01	1.05	***
Maternal Race (Reference: Non-Hispanic Non-Black)								
Hispanic	-0.10	0.03	0.90	**	-0.14	0.03	0.87	***
Black	-0.21	0.03	0.81	***	-0.25	0.04	0.78	***
Maternal Age at Offspring Birth	-0.06	0.00	0.94	***	-0.06	0.00	0.94	***
Maternal Marital Status as Offspring Birth (Reference: Married)								
Cohabiting	0.11	0.05	1.12	*	0.08	0.05	1.09	*
Single	0.00	0.03	1.00		-0.02	0.03	0.98	
Percentage of Childhood Exposed to Marriage	-0.01	0.04	0.99		0.04	0.05	1.04	
Percentage of Childhood Exposed to Cohabitation	0.13	0.08	1.14		0.11	0.08	1.12	
Maternal Education (Reference: High School)								
Less than High School					0.05	0.04	1.05	
Some College					0.02	0.03	1.02	
College					-0.14	0.06	0.87	***
More than College					-0.15	0.08	0.86	**
Percentage of Childhood in Poverty					0.22	0.09	1.24	***
Percentage of Childhood Mother Worked Full Time					0.10	0.07	1.11	*
Percentage of Childhood Mother Worked Part Time					0.05	0.07	1.05	
Offspring Male	0.13	0.02	1.14	***	0.13	0.02	1.14	***
Offspring is a Parent	0.72	0.03	2.06	***	0.69	0.04	2.00	***
*F*	214.67***				117.66***			

Note

* *p* < .05.

** *p* < .01

*** *p* < .001.

IRR = Incident Rate Ratio. Model controls for exposure (offspring age). Standard errors adjusted for clustering by mother.

**Table 3:** Results of population poisson models with maternal partners separated by cohabiting and married.

	Partner Type, no SES				Partner Type, SES			
	*b*	*SE*	*IRR*		*b*	*SE*	*IRR*	
Maternal Marital Partners	0.07	0.02	1.07	***	0.07	0.02	1.07	***
Maternal Cohabiting Partners	0.04	0.01	1.04	***	0.03	0.01	1.03	**
Maternal Race (Reference: Non-Hispanic Non-Black)								
Hispanic	-0.09	0.03	0.91		-0.14	0.03	0.87	***
Black	-0.20	0.03	0.82	*	-0.24	0.04	0.79	***
Maternal Age at Offspring Birth	-0.06	0.00	0.94	***	-0.06	0.00	0.94	***
Maternal Marital Status as Offspring Birth (Reference: Married)								
Cohabiting	0.10	0.05	1.10	*	0.07	0.05	1.08	
Single	0.00	0.03	1.00		-0.02	0.03	0.98	
Percentage of Childhood Exposed to Marriage	-0.03	0.04	0.97		0.02	0.05	1.02	
Percentage of Childhood Exposed to Cohabitation	0.16	0.09	1.17	*	0.14	0.09	1.15	*
Maternal Education (Reference: High School)								
Less than High School					0.05	0.04	1.05	
Some College					0.01	0.03	1.01	
College					-0.14	0.06	0.87	***
More than College					-0.15	0.08	0.86	**
Percentage of Childhood in Poverty					0.20	0.10	1.23	***
Percentage of Childhood Mother Worked Full Time					0.08	0.07	1.08	
Percentage of Childhood Mother Worked Part Time					0.03	0.07	1.03	
Offspring Male	0.13	0.02	1.14	***	0.13	0.02	1.14	***
Offspring is a Parent	0.72	0.03	2.06	***	0.69	0.04	2.00	***
*F*	199.26***				114.30***			

Note

* *p* < .05.

** *p* < .01

*** *p* < .001.

IRR = Incident Rate Ratio. Model controls for exposure (offspring age). Standard errors adjusted for clustering by mother.

### Sibling model results

To test our competing second and third hypotheses, we examined sibling differences in the association between maternal and offspring partnering using hybrid sibling Poisson regression models (see Table 4). Between-family effects, displayed in the top of the table, corroborated the pooled models; an increase in maternal partners was associated with a 5% greater incident rate of offspring partners. However, the within-family effects, reported in the bottom of the table, suggested that siblings were not significantly different from one another (*p* = .08). Findings by type of maternal partners were similar to the pooled results between mothers: between-family effects indicated that both marital and cohabiting partners were significantly associated with more offspring partners, but neither were associated with offspring instability within families. Between family-effects of the control variables were similar to the clustered Poisson regression models in that maternal race, maternal age at childbirth, maternal college education, childhood poverty, and offspring gender and parental status were all significantly associated with offspring partnering. The within-family effects show that older siblings (that is, siblings born to younger mothers) had significantly more partners, as did brothers compared to sisters and siblings who were parents. Additionally, siblings exposed to cohabitation for more of their childhoods reported more partners. Models by partner type replicated these findings.

**Table 4:** Results of hybrid sibling poisson models, various partner specifications.

	Total Partners				Partner Type			
Between Family Effects:	*b*	*SE*	*IRR*		*b*	*SE*	*IRR*	
Maternal Partners (Total)	0.05	0.01	1.05	***				
Maternal Marital Partners					0.06	0.02	1.06	**
Maternal Cohabiting Partners					0.04	0.01	1.04	**
Maternal Race (Reference: Non-Hispanic Non-Black)								
Hispanic	-0.16	0.03	0.86	***	-0.15	0.03	0.86	***
Black	-0.29	0.04	0.75	***	-0.28	0.04	0.76	***
Maternal Age at Offspring Birth	-0.07	0.00	0.94	***	-0.06	0.00	0.94	***
Maternal Marital Status as Offspring Birth (Reference: Married)							
Cohabiting	-0.12	0.07	0.89		-0.11	0.07	0.90	
Single	-0.09	0.07	0.91		-0.07	0.07	0.93	
Percentage of Childhood Exposed to Marriage	0.02	0.05	1.02		0.00	0.05	1.00	
Percentage of Childhood Exposed to Cohabitation	0.06	0.09	1.06		0.08	0.10	1.08	
Maternal Education (Reference: High School)								
Less than High School	0.01	0.04	1.01		0.01	0.04	1.01	
Some College	0.03	0.03	1.03		0.03	0.03	1.03	
College	-0.13	0.07	0.88	*	-0.13	0.07	0.88	*
More than College	-0.10	0.08	0.91		-0.10	0.08	0.91	
Percentage of Childhood in Poverty	0.21	0.10	1.23	*	0.20	0.10	1.22	*
Percentage of Childhood Mother Worked Full Time	0.12	0.07	1.12		0.10	0.07	1.10	
Percentage of Childhood Mother Worked Part Time	0.06	0.07	1.06		0.04	0.07	1.05	
Male Offspring	0.13	0.03	1.13	***	0.78	0.04	2.17	***
Parental Status	0.78	0.04	2.17	***	0.78	0.04	2.17	***
Within Family Effects:								
Maternal Partners (Total)	0.10	0.05	1.10					
Maternal Marital Partners					0.12	0.07	1.12	
Maternal Cohabiting Partners					0.06	0.08	1.06	
Maternal Age at Offspring Birth	-0.07	0.01	0.93	***	-0.07	0.01	0.93	***
Maternal Marital Status as Offspring Birth (Reference: Married)							
Cohabiting	-0.09	0.08	0.91		-0.08	0.08	0.92	
Single	-0.15	0.08	0.86		-0.14	0.09	0.87	
Percentage of Childhood Exposed to Marriage	0.26	0.18	1.30		0.25	0.18	1.29	
Percentage of Childhood Exposed to Cohabitation	0.87	0.31	2.39	**	0.84	0.31	2.31	**
Maternal Education (Reference: High School)								
Less than High School	0.13	0.13	1.14		0.13	0.13	1.14	
Some College	-0.14	0.13	0.87		-0.14	0.13	0.87	
College	0.17	0.23	1.19		0.18	0.23	1.19	
More than College	-0.05	0.34	0.95		-0.06	0.34	0.94	
Percentage of Childhood in Poverty	0.15	0.14	1.16		0.15	0.14	1.16	
Percentage of Childhood Mother Worked Full Time	0.02	0.25	1.02		-0.01	0.25	0.99	
Percentage of Childhood Mother Worked Part Time	0.04	0.27	1.04		0.03	0.27	1.03	
Offspring Male	0.15	0.03	1.16	***	0.55	0.04	1.73	***
Offspring is a Parent	0.55	0.04	1.73	***	0.55	0.04	1.73	***
*F*	49.83***				47.95***			

Note

* *p* < .05

** *p* < .01

*** *p* < .001.

IRR = Incident Rate Ratio. Model controls for exposure (offspring age) and sibling order.

## Discussion

Half of first cohabitations will dissolve within three years, and half of first marriages will dissolve within twenty years [2, 3]. Those who experience union dissolution often repartner [4], potentially having children with new partners [70]. Thus, family complexity continues to increase in the United States [71]. Our results suggest that offspring whose mothers have multiple partners will be significantly more likely to have multiple partners themselves. We extended research by Amato and Patterson [18] that found an intergenerational transmission of family instability by focusing on partnering using prospective maternal data on union formation and economic instability and exploiting sibling data in the NLSY79 and NLSY79 CYA.

We outlined three potential perspectives that could explain the intergenerational transmission of partnering. The economic hardship perspective suggested that the economic stress that often accompanies union instability [27] contributes to greater offspring union instability and thus increased partnering. Our results confirmed that childhood socioeconomic vulnerability contributed to offspring partnering. Offspring who had more exposure to poverty reported significantly more partners whereas offspring who had mothers with more education reported fewer. Interestingly, offspring whose mothers worked full-time actually reported more partners than those whose mothers did not work. Perhaps due to a lack of policy supports for working families in the US, such as paid maternity leave, working mothers in the US are more likely to divorce than those in other countries [72], increasing the risk of union dissolution for their own offspring. Although socioeconomic factors were significantly associated with partnering, they did not significantly reduce the association between maternal partnering and offspring partnering, suggesting that the economic instability associated with partner transitions did not explain the intergenerational transmission of partnering.

The transmission of commitment perspective [14] suggested that offspring who observed their mother exiting marital and cohabiting unions, perhaps multiple times, learn that commitments can be broken and that new partnerships can be formed that may be more beneficial for the individual [11]. According to this perspective, witnessing the dissolution itself is the mechanism driving increased partnering among offspring [14]. Based on this perspective, we expected that siblings who experienced different levels of maternal partnering would differ from one another such that a sibling who experienced greater maternal partnering would be more likely to experience more partnering compared to siblings who experienced less maternal partnering. We did not find a significant differential sibling effect, and thus we did not find specific evidence in support of the transmission of commitment perspective.

Our pattern of results most strongly supported the intergenerational transmission of marriageable characteristics and relationship skills perspective. This perspective suggested that mothers have certain characteristics that make them more or less desirable on the marriage market and better or worse at relationships [21], and children inherit and learn these skills and behaviors which they then take with them into their own intimate relationships [14, 73]. Offspring who experience poorer parental relationships may lack positive relationship skills, rendering their own relationships less stable. Using sibling models to account for sibling invariant maternal characteristics, we found that siblings reported similar levels of partnering even if they differed in their experience of their mothers’ partnering. For example, a sibling who experienced their mother moving from a first union into a second did not have a statistically greater number of partners compared to their half sibling who was born in their mother’s second union. The overall findings thus support the assertion that the mechanism underlying the intergenerational transmission of partnering may be the intergenerational transmission of marriageable characteristics and relationship skills.

There is some evidence that the commitment perspective should be considered in future research. Although not statistically significant, the association between maternal partnering and within-family offspring partnering was positive and larger in magnitude than the between-family effect. To further test the notion that maternal models of commitment may also play a role in these findings, we tested differences in offspring exposure to cohabitation versus marriage, both by the number of partners of each type the offspring witnessed and by the percent of their childhood their mother was in each type of union. Because cohabiting unions are most often less committed than marital unions [57] and often short lived [2], greater exposure to maternal cohabitations was hypothesized to be more strongly associated with offspring partnering than maternal marriage. We found no within-family (sibling) differences by type of maternal partner; rather, we found that experiencing more maternal cohabiting and marital partners were similarly associated with greater offspring partnering. We did find that siblings exposed to cohabitation longer experienced more partnering than their siblings exposed to less cohabitation. Offspring exposed to cohabitation for longer durations may come to view cohabitation as an attractive, lower-commitment union, which may open the door to repartnering given cohabitation’s high dissolution rates [2].

### Limitations

The NLSY79 and NLSY79 CYA datasets contain a wealth of information, yet have limitations. First, the sample of mothers in the NLSY79 was nationally representative when data collection began, but United States demographics have changed dramatically over the years and these data no longer mirror demographics of the nation today. Second, the NLSY79 undercounts maternal partnerships. Although participants could report themselves as being in an unmarried partnership at the time of the survey each year, cohabiting unions that occurred between surveys were not measured until later; thus, cohabitations were difficult to track and were underestimated. Third, these data lack consistent measurement of mechanistic variables such as relationship quality, stress, commitment, and mental health, which would have allowed us to explore directly the transmissions of marriageable traits, relationship skills, and commitment, rather than theoretically speculate as to the mechanisms. Future research should more closely examine the association between mother and offspring’ characteristics and behaviors their association with union formation and dissolution. Further, longitudinal data on marriageable traits and relationship skills, as well as commitment, would allow us to examine whether these characteristics are consistent across unions, and across the adulthood years. It is possible that relationship skills change from union to union, for example. Fourth, paternal data were unavailable. Limiting data to maternal households fails to fully capture family complexity [74]. Fifth, single mothers can have difficulty partnering into unions [75] and may instead maintain sexual and dating relationships outside of coresidence. When single mothers do date, their children may be involved in the dating process [76]. Thus, children of single mothers may also witness their mother interacting with her sexual and dating partners, which may further shape their perceptions of relationship functioning. These data do not have consistent data on sexual and dating relationships that occurred outside of unions.

Right-censoring was also a threat to validity because offspring have not yet completed partnering. We controlled for offspring age, but additional data from further into the life course may show a different pattern of results. A critique of sibling models is that parents may deliberately attempt to make siblings similar (compensation) or different (selective investment) from one another [77]. If compensation or selective investment were occurring in our sample and the siblings were either artificially homogeneous or heterogeneous, the results of the hybrid sibling fixed-effects model may be biased. These data did not allow us to assess compensation or selective investment efforts. Another critique of sibling models is that these models do not account for any influence that siblings may have on each other, which would likely lead siblings to be more homogeneous than they would be without this influence. Data on sibling relationships, including contact, closeness, and even data on how siblings talk about relationships [78] may suggest additional mechanisms underlying the intergenerational transmission of partnering. Finally, unobservable characteristics that are shared between siblings, other than those outlined by the transmission of marriageable characteristics and relationship skills perspective, could also be responsible for the transmission of partnering. For example, our measures of economic hardship were limited. The intergenerational transmission of parental wealth and socioeconomic status may also be driving our results.

Finally, as noted in the introduction, partnering encompasses a number of different behaviors, including an individual’s choice to form a first union, dissolve or divorce a union, and form a new union afterwards. Future work would do well to isolate the various components of partnering shared by mothers and offspring to better understand the specific behaviors being passed through generations.

## Conclusion

We found that partnering was transmitted across generations in our sample of mothers and their young adult offspring [18], even after accounting for prospective measures of economic instability as well as sibling differences in partnering. Our findings suggest that the most plausible mechanism underlying the intergenerational transmission of partnering may be the transmission of poor marriageable characteristics and relationship skills, which can include but are not limited to conflict resolution skills, personality, and mental health, although future research should measure and test these characteristics directly. Poor marriageable characteristics such as personality traits may actually be malleable [79] and the clinical psychological literature has consistently shown that relationship skills can be improved [80]. Yet the longevity of the benefits of relationship interventions is up for debate [81] and these interventions appear to have less utility for couples that are not white and middle-class [82]. If Finkel and Hui [83] are correct and American’s expectations of their intimate relationships are at an all-time high, strategies to improve relationship skills may be of greater importance now than ever before because relationship expectations are so hard to meet. On the other hand, if young adults today are pruning their unions through serial monogamy [84] until they find a suitable partner with compatible marriageable characteristics and relationship skills, we may see increased union stability in later unions. Future research on current cohorts of young adults will shed much needed light on contemporary family demography.

## Supporting information

S1 File Variables from NLSY79 CYA cohort used to create offspring variables. (CHILDYA)

S2 File Variables from NLSY79 cohort used to create maternal covariates. (NLSY79)

S3 File Variables from NLSY79 cohort used to create maternal partnering history. (NLSY79)
